# Infection risk associated with teclistamab in relapsed/refractory multiple myeloma: a systematic review and meta-analysis of clinical trial and real-world evidence

**DOI:** 10.3389/fimmu.2026.1804838

**Published:** 2026-04-30

**Authors:** Zeng-Yi Huang, Jialiang Chen, Boman Zhu, Xiao-Lian Liu

**Affiliations:** Department of Hematology, Gaozhou People’s Hospital, , Maoming, Guangdong, China

**Keywords:** bispecific antibodies, infection, meta-analysis, multiple myeloma, real-world evidence, teclistamab

## Abstract

**Background:**

Teclistamab, a B-cell maturation antigen (BCMA) × CD3 bispecific antibody (BsAb), has shown remarkable efficacy in relapsed/refractory multiple myeloma (RRMM). However, its mechanism leads to profound hypogammaglobulinemia, making infection a critical concern. This systematic review and meta-analysis aimed to quantify the infectious burden and contrast outcomes between clinical trial and real-world evidence (RWE).

**Methods:**

We systematically searched PubMed, Embase, Web of Science, and the Cochrane Library for studies reporting infection outcomes in RRMM patients treated with teclistamab. Pooled incidences of any-grade and grade ≥3 infections were calculated using a random-effects model. Subgroup analysis compared the pivotal MajesTEC-1 trial with multi-institutional RWE cohorts.

**Results:**

Five studies encompassing 714 patients were included. The overall pooled incidence was 56.5% (95% CI: 43.1%–69.9%) for any-grade infections and 27.6% (95% CI: 21.0%–34.3%) for grade ≥3 infections. Subgroup analysis revealed a significantly higher risk in the clinical trial compared to RWE (Any-grade: 76.4% vs. 45.4%, p< 0.01; Grade ≥3: 44.8% vs. 22.8%, p<0.01). Infection-related mortality was reported in all cohorts, ranging from 0.9% to 7.3%, with COVID-19 and opportunistic pathogens (for example, Pneumocystis jirovecii) being prevalent. Significant heterogeneity was driven by variations in follow-up duration and intravenous immunoglobulin (IVIG) prophylaxis rates (range: 41.8%–81.3%).

**Conclusions:**

Teclistamab is associated with a substantial and cumulative infectious burden. The lower infection rates in RWE may reflect shorter follow-up and evolving prophylactic strategies. Standardized infection surveillance, including regular IgG monitoring and consideration of IVIG replacement in patients with low IgG levels, may help optimize the safety of BCMA-directed bispecific therapies.

**Systematic Review Registration:**

https://www.crd.york.ac.uk/prospero/, identifier CRD420261297645.

## 1 Introduction

The therapeutic landscape for relapsed/refractory multiple myeloma (RRMM) has been revolutionized by the emergence of therapies targeting the B-cell maturation antigen (BCMA) (1). Among these innovations, teclistamab, a first-in-class BCMA × CD3 bispecific antibody (BsAb), has demonstrated significant clinical efficacy, offering deep and durable responses in heavily pretreated patients who have exhausted standard-of-care options (2, 3). By simultaneously binding to CD3 on T cells and BCMA on myeloma cells, teclistamab induces T-cell activation and subsequent lysis of malignant plasma cells (2).Unlike certain therapies administered for a fixed duration, teclistamab is typically administered as a continuous therapy until disease progression or unacceptable toxicity.

However, the potent anti-tumor activity of teclistamab comes at the cost of profound immune dysregulation (4, 5). Because BCMA is also expressed on healthy plasma cells and late-stage B cells, teclistamab therapy leads to “on-target, off-tumor” effects, characterized by continuous B-cell depletion and rapid-onset hypogammaglobulinemia (4, 6). This creates a state of severe secondary humoral immunodeficiency, significantly increasing the patient’s susceptibility to a wide spectrum of infectious complications, ranging from common bacterial pathogens to rare opportunistic infections (5, 7).

Recent clinical reports and early systematic reviews have identified infection as the leading cause of non-hematologic toxicity and a primary driver of treatment interruption and mortality in patients receiving teclistamab (2, 5, 8). While several meta-analyses have provided broad safety overviews, they often aggregate all toxicities or rely predominantly on early-phase trial data with limited follow-up (9, 10). Furthermore, as teclistamab moves into standard clinical practice, emerging real-world evidence (RWE) has suggested potential discrepancies in infection rates and management strategies compared to the controlled MajesTEC-1 trial environment (8, 11).

Currently, a dedicated and up-to-date meta-analysis that integrates both pivotal trial data and multi-institutional real-world evidence to specifically evaluate the infection burden of teclistamab is missing. Therefore, we conducted this systematic review and meta-analysis to quantify the pooled incidence of any-grade and grade ≥3 infections, explore the differences between trial and real-world settings, and provide evidence-based insights for infectious disease management in the era of bispecific antibodies (5, 7). In contrast to prior safety meta-analyses that pooled heterogeneous immune-related adverse events or relied predominantly on early-phase trial data, the present study exclusively focuses on infection outcomes and uniquely integrates both pivotal trial data and contemporary multi-center real-world cohorts. This design allows for a more clinically relevant estimation of infection risk under routine practice conditions.

## 2 Methods

This systematic review and meta-analysis was conducted in accordance with the Preferred Reporting Items for Systematic Reviews and Meta-Analyses (PRISMA) 2020 statement (12). The study protocol was prospectively registered with PROSPERO (registration number: CRD420261297645) (13).

### 2.1 Search strategy

We conducted a comprehensive systematic search of the PubMed, Embase, Web of Science, and the Cochrane Library databases from inception through January 2026. The search strategy employed a combination of Medical Subject Headings (MeSH) terms and free-text keywords, including “teclistamab,” “JNJ-64007957,” “multiple myeloma,” and “infection.” No language restrictions were applied. To ensure study completion, the reference lists of relevant reviews and the identified studies were manually screened for additional eligible citations.

### 2.2 Inclusion and exclusion criteria

Studies were included if they met the following criteria: (1) involved patients with relapsed/refractory multiple myeloma (RRMM) treated with teclistamab; (2) reported the incidence of infections, specifically categorized by severity (any-grade and grade ≥3); and (3) were either clinical trials or original real-world studies (cohort studies or case series). Exclusion criteria were: (1) studies with overlapping patient populations (in which case, the report with the longest follow-up or largest sample size was selected); (2) case reports, editorials, and review articles; and (3) studies with insufficient data for calculating pooled incidence rates.

### 2.3 Data extraction and management

Two independent reviewers extracted data from the included studies using a standardized form. The following information was collected: first author, year of publication, study design (Trial vs. RWE), sample size (N), median age, median prior lines of therapy, and the number of events (n) for any-grade and grade ≥3 infections. Any discrepancies between the reviewers were resolved through consensus or consultation with a third senior author.

### 2.4 Quality assessment

Since the majority of the included studies were single-arm clinical trials or retrospective cohort studies, the methodological quality was assessed using the Joanna Briggs Institute (JBI) Critical Appraisal Checklist for Case Series (14). This tool evaluates 10 domains, including inclusion criteria, measurement of the condition, and reporting of clinical information. Studies were categorized as good, fair, or poor quality based on the total number of “Yes” responses.

### 2.5 Statistical analysis

All statistical analyses were performed using the meta package (version 7.0 or higher) in R software (version 4.3.0) (15).

Pooled incidence: The pooled incidences of any-grade and grade ≥3 infections were calculated using a random-effects model to account for anticipated inter-study heterogeneity (16).

Heterogeneity assessment: Heterogeneity refers to variability in study outcomes beyond what would be expected by chance alone. It was quantified using the I² statistic and Cochran’s Q test (17, 18). I^2^ values of 25%, 50%, and 75% were considered to represent low, moderate, and high heterogeneity, respectively.

Subgroup and sensitivity analysis: To explore sources of heterogeneity, subgroup analyses were conducted based on study design (Trial vs. Real-world evidence). Furthermore, a leave-one-out sensitivity analysis was performed by sequentially removing each study to evaluate the stability and robustness of the overall pooled estimates.

Publication bias: Publication bias refers to the preferential publication of studies with positive or statistically significant findings, which may distort pooled estimates. Publication bias was assessed using Egger’s regression test and visual inspection of funnel plots when at least 10 studies were included, in accordance with Cochrane recommendations.

## 3 Results

### 3.1 Study selection

**Figure 1:**
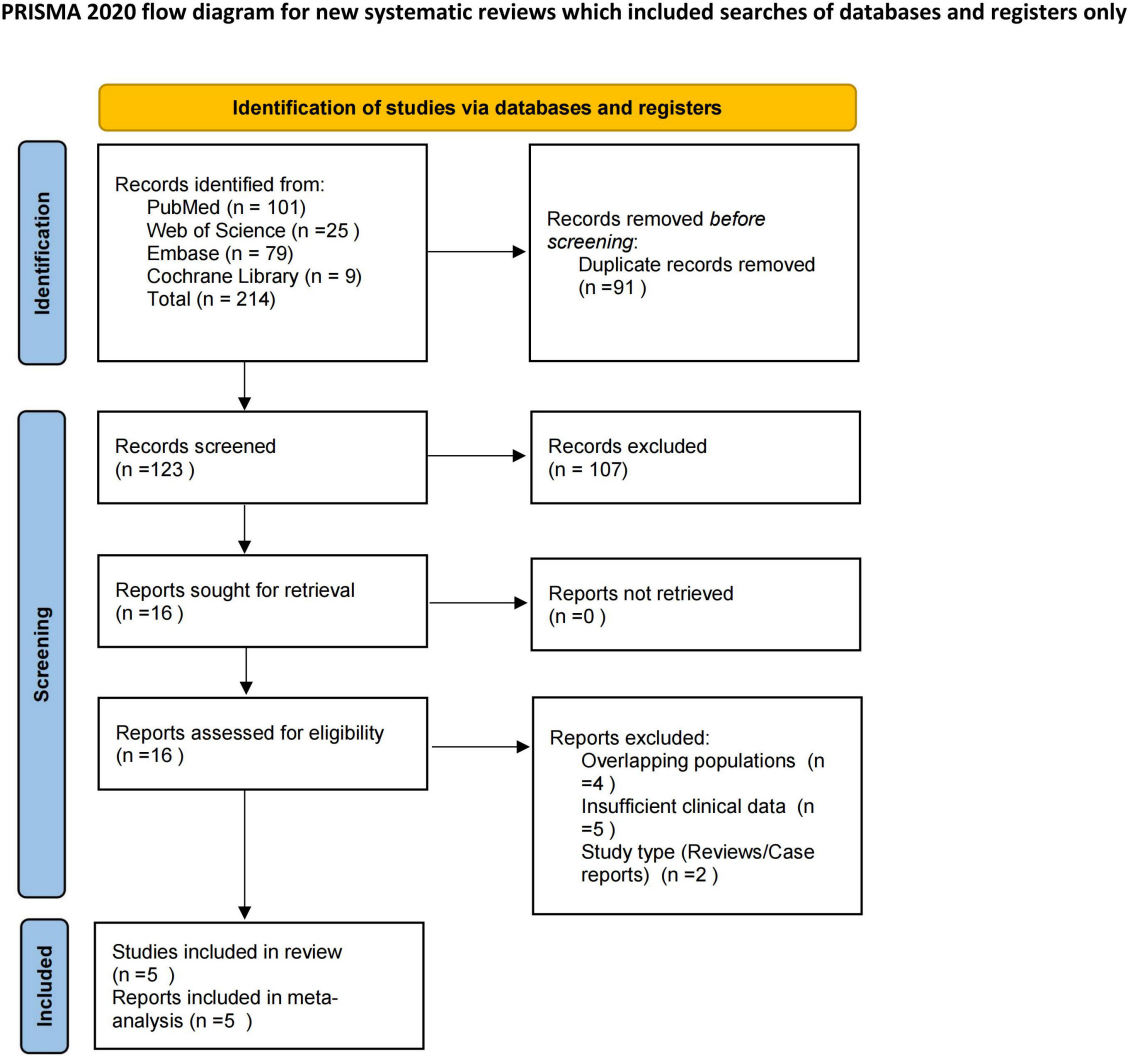
PRISMA 2020 flow diagram of the study selection process. The flowchart illustrates the systematic identification, screening, and inclusion of studies. From 214 initial citations, 5 studies (1 clinical trial and 4 real-world studies) involving 714 patients were ultimately included in the meta-analysis.

The initial database search yielded 214 citations. After removing 91 duplicates, 123 records were screened based on titles and abstracts. Sixteen full-text articles were assessed for eligibility, of which 11 were excluded (4 for overlapping cohorts, 5 for insufficient clinical data, and 2 for study type). Finally, 5 studies (1 clinical trial and 4 real-world studies) met all inclusion criteria and were included in this meta-analysis. The detailed screening process is illustrated in the PRISMA flow diagram (**Figure 1**).

### 3.2 Study characteristics and quality assessment

The 5 included studies encompassed a total of 714 patients with RRMM treated with teclistamab (2, 8, 11, 19, 20). The baseline characteristics are summarized in **Table 1**. Most patients were heavily pretreated, with a median of 5–6 prior lines of therapy. Notably, while the MajesTEC-1 trial excluded patients with prior BCMA-targeted therapy (2), real-world studies included significant proportions of BCMA-exposed patients (ranging from 35.5% to 53.0%) (8, 11, 19, 20).

**Table 1:** Baseline characteristics of the included studies.

Characteristic	Moreau et al., 2022 (2)	Tan et al., 2025 (20)	Riedhammer et al., 2024 (11)	Mohan et al., 2024 (8)	Dima et al., 2024 (19)
Study Design	Phase 1–2 trial (MajesTEC-1)	Multicenter retrospective RWE	Multicenter retrospective RWE	Multi-institutional retrospective RWE	Multi-institutional retrospective RWE
Sample Size (N)	165	210	123	110	106
Age, median (range)	64 (33–84)	67 (33–91)	67 (35–87)	68 (37–89)	66.5 (35–87)
Gender (Male/Female)	96/69	117/93	70/53	56/54	49/57
Prior lines, median (range)	5 (2–14)	6 (1–20)	6 (3–14)	6 (3–13)	6 (4–17)
Triple-class refractory, n (%)	165 (100)	138/167 (82.6)	114 (92.7)	95 (86)	97 (91.5)
Penta-drug refractory, n (%)	116 (70.3)	71/161 (44.1)	74 (60.2)	84 (76)	68 (64)
Prior BCMA therapy, n (%)	0 (0)	92 (43.8)	46 (37.4)	39 (35.5)	56 (53)
Median follow-up (months)	14.1	5.3	5.5	3.5	3.8

RWE, real-world evidence; BCMA, B-cell maturation antigen.

Summary of patient demographics and clinical history. Notably, the median number of prior therapy lines was 5–6, and the proportion of prior BCMA-targeted therapy ranged from 0% in the pivotal trial to 53% in RWE cohorts.

**Figure 2:**
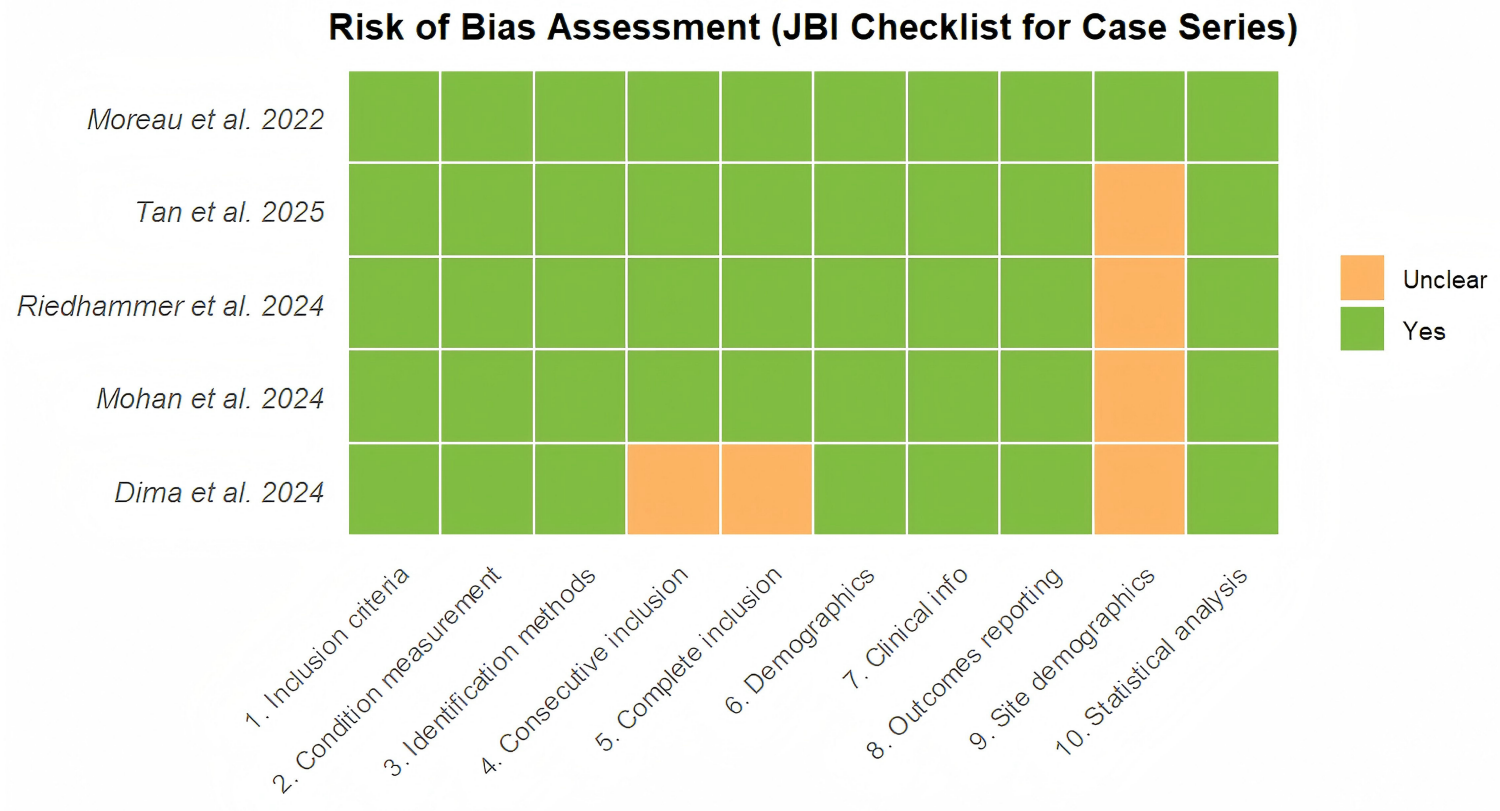
Risk of bias summary plot (traffic-light format). Methodological quality assessment of the 5 included studies using the JBI Critical Appraisal Checklist for Case Series. Green circles indicate “Yes” (low risk), and yellow circles indicate “Unclear” (potential risk). Domain 9 (site demographics) was a common source of unclear risk in real-world cohorts.

Methodological quality assessment using the JBI Checklist indicated that 4 studies were of “Fair” quality and 1 study (Moreau 2022) was of “Good” quality (**Supplementary Table 1**) (2). The primary sources of bias identified were the lack of a control group and relatively short follow-up durations in real-world cohorts. The risk of bias assessment is visualized in the traffic light plot (**Figure 2**).

### 3.3 Primary outcomes: incidence of infection

The pooled incidences and specific infectious complications across all included studies are summarized in **Table 2**.

**Table 2:** Summary of infection outcomes and safety across included studies.

Outcomes	Moreau et al., 2022 (2)	Tan et al., 2025 (20)	Riedhammer et al., 2024 (11)	Mohan et al., 2024 (8)	Dima et al., 2024 (19)
Infection Incidence					
Any-grade, n (%)	126 (76.4)	118 (56.2)	67 (54.5)	44 (40.0)	33 (31.1)
Grade ≥3, n (%)	74 (44.8)	46 (21.9)	33 (26.8)	29 (26.4)	18 (17.0)
Mortality					
Infection-related, n (%)	12 (7.3)	7 (3.3)	5 (4.1)	1 (0.9)	1 (0.9)
Common Pathogens (Incidence %)	Bacterial: 39.4%; Viral: 52.1% (COVID-19: 17.6%); Fungal: 0.6%; PJP: 1.2% (2/165)	Bacterial: 52%; Viral: 40%; Fungal: 8%	Bacterial and Fungal (Proportions NR)	Bacterial: 48% (Bacteremia 11%); Viral: 45% (COVID-19 10%); Fungal: 6.7% (PJP 4%); Respiratory: 44.6%	Bacterial: 41%; Viral: 51% (COVID-19, Rhino/Adenovirus); Fungal: 3%; Respiratory: 69%
Prophylaxis					
IVIG use, n (%)	NR	129 (61.4)	100 (81.3)	46 (41.8)	52 (49.0)
PJP/HSV prophylaxis	Mandatory	Routine	Routine	Routine	Routine

NR, not reported; IVIG, intravenous immunoglobulin; PJP, Pneumocystis jirovecii pneumonia; HSV, herpes simplex virus.

This table quantifies any-grade and grade ≥3 infection rates, infection-related mortality (0.9%–7.3%), and the distribution of major pathogens (COVID-19, bacterial pneumonia, and PJP). Intravenous immunoglobulin (IVIG) utilization rates across cohorts are also summarized.

**Figure 3:**
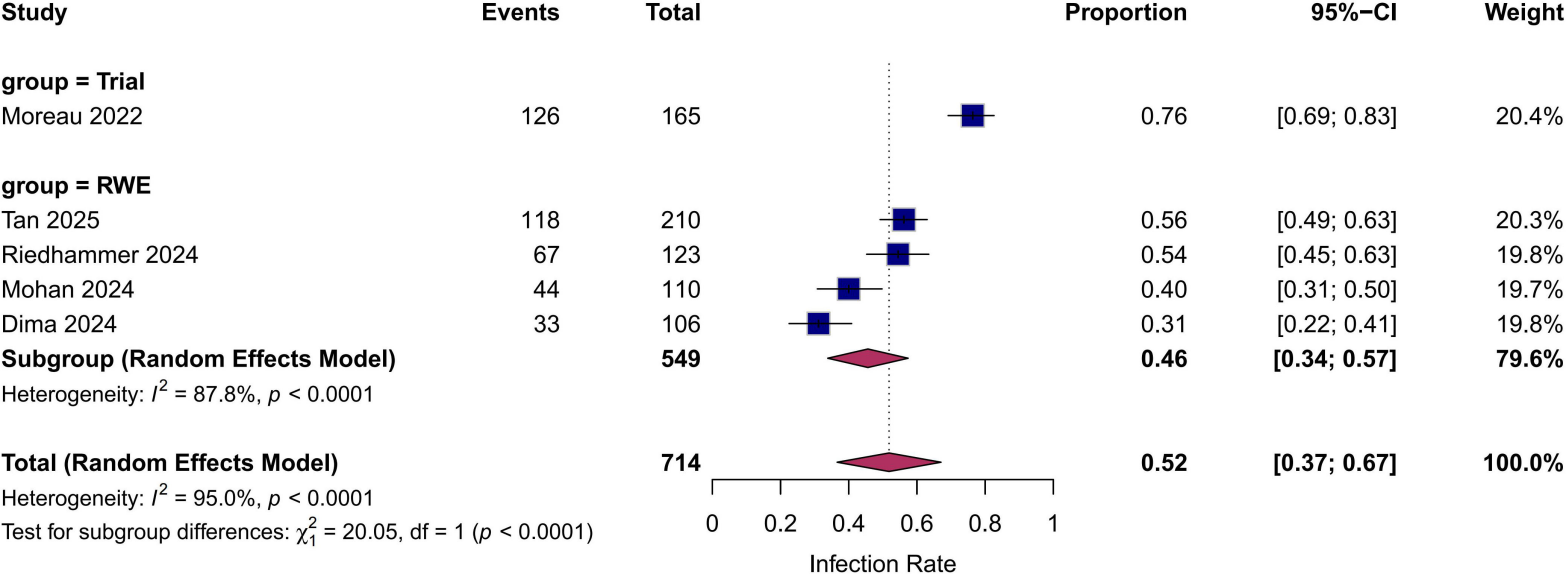
Forest plot of the pooled incidence of any-grade infections. Pooled analysis showing an overall incidence of 56.5% (95% CI: 43.1%–69.9%). Subgroup analysis reveals a significantly higher incidence in the clinical trial (76.4%) compared with the real-world evidence (RWE) subgroup (45.4%; p<0.01 for subgroup difference). I² indicates high heterogeneity.

Any-grade infection: The pooled incidence was 56.5% (95% CI: 43.1%–69.9%; **Figure 3**). Significant heterogeneity was observed (I^2^ = 89%, p<0.01).

**Figure 4:**
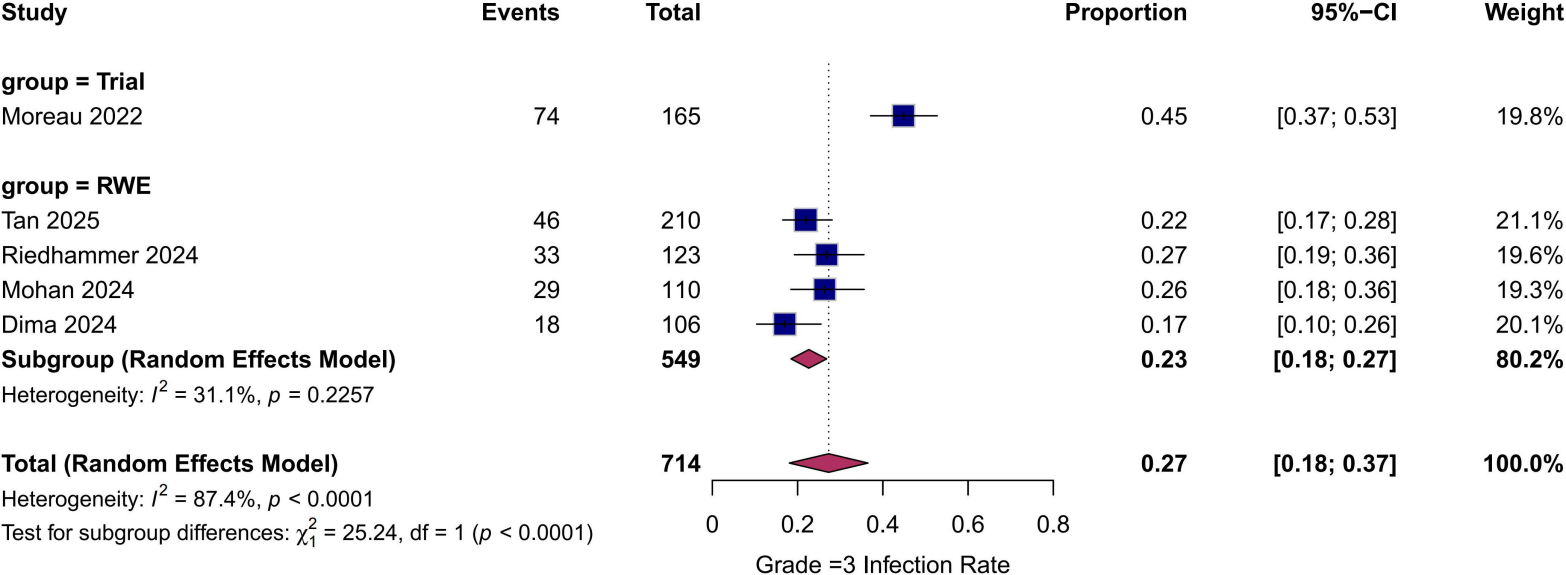
Forest plot of the pooled incidence of grade ≥3 infections. The overall incidence of severe (grade ≥3) infections was 27.6% (95% CI: 21.0%–34.3%). The trial reported a higher rate (44.8%) than the pooled RWE cohorts (22.8%; p<0.01). Subgroups are analyzed using a random-effects model.

Grade ≥3 infection: The pooled incidence of severe infection was 27.6% (95% CI: 21.0%–34.3%; **Figure 4**). Heterogeneity remained substantial (I^2^ = 78%, p<0.01).

Heterogeneity explanation: The observed high heterogeneity may be partially attributed to variations in baseline clinical characteristics, particularly the higher proportion of prior BCMA-targeted therapy exposure and shorter median follow-up durations (3.5–5.5 months) in RWE cohorts compared to the pivotal trial.

### 3.4 Subgroup analysis: trial vs. real-world evidence

To further explore heterogeneity, subgroup analyses were performed by study design. The any-grade infection rate was significantly higher in the clinical trial subgroup (76.4%) than in the RWE subgroup (pooled incidence: 45.4%, 95% CI: 35.2%–55.7%; P for subgroup difference < 0.01; **Figure 3**). Similarly, for grade ≥3 infections, the trial reported a higher incidence (44.8%) compared to the pooled RWE incidence (22.8%, 95% CI: 18.3%–27.3%; p<0.01; **Figure 4**).

### 3.5 Sensitivity analysis

A leave-one-out sensitivity analysis confirmed the robustness of the findings. For both any-grade and grade ≥3 infections, the sequential removal of any single study did not significantly alter the pooled estimates, which remained within the 95% CI of the primary analysis (**Supplementary Figures 1**, **S2**). This indicates that the results were not disproportionately influenced by the high-incidence MajesTEC-1 trial.

### 3.6 Infection-related mortality

Detailed safety outcomes are presented in **Table 2**. Fatal infectious events (Grade 5) were reported in all cohorts. In the MajesTEC-1 trial, 7.3% (12/165) of deaths were attributed to infections, primarily COVID-19 (2). In RWE cohorts, infection-related mortality ranged from 0.9% to 4.1%. While seemingly lower than trial data, this discrepancy likely reflects the significantly shorter follow-up duration in real-world settings and potential under-reporting of late-onset infectious complications. Major pathogens identified included bacterial pneumonia, COVID-19, and opportunistic fungal infections such as Pneumocystis jirovecii pneumonia (PJP) (8, 19). Collectively, these findings underscore that while teclistamab offers deep hematological responses, infection remains a significant lethal risk requiring vigilant management, especially as therapy durations extend in routine clinical practice.

## 4 Discussion

This systematic review and meta-analysis provides a comprehensive quantification of the infection burden associated with teclistamab in patients with relapsed/refractory multiple myeloma (RRMM). By integrating the pivotal MajesTEC-1 trial with emerging multi-institutional real-world evidence (N = 714), our study demonstrates a high pooled incidence of any-grade (56.5%) and grade ≥3 (27.6%) infections (2, 8, 11, 19, 20). Importantly, these findings highlight a clinically meaningful discrepancy between infection rates reported in controlled trial settings and those observed in routine practice, while underscoring the urgent need for standardized and proactive infection prevention strategies.

One of the most striking observations in our analysis is the substantially higher incidence of infections reported in the MajesTEC-1 trial (76.4%) compared with pooled real-world cohorts (45.4%) (2). Several factors may account for this apparent gap between efficacy and safety. First, the intensive monitoring protocols inherent to clinical trials, including frequent laboratory testing and systematic adverse event reporting, are likely to increase the detection of low-grade infections that may be underrecognized in real-world practice. Second, the disparity in follow-up duration warrants critical attention. Patients in the MajesTEC-1 trial had a median follow-up of 14.1 months (2), whereas most RWE cohorts reported significantly shorter periods (typically 3.5–5.5 months) (8, 11, 19, 20). Given the continuous dosing schedule of teclistamab, infection risk is cumulative rather than transient. It is highly probable that as real-world follow-up extends, the reported infection rates will climb and eventually converge with clinical trial data, suggesting that the current RWE data may represent an underestimation of the long-term infectious toll.

The substantial heterogeneity observed across studies reflects the complex interplay of biological and system-level variables. A pivotal factor contributing to this variance is the inconsistent implementation of IVIG replacement therapy. Our analysis (**Table 2**) revealed a vast range of IVIG utilization across studies, from as low as 41.8% to as high as 81.3% (8, 11, 19, 20). This heterogeneity in prophylactic intensity likely serves as a primary driver of the differing infection rates observed. For instance, studies with higher IVIG uptake generally reported a more manageable incidence of severe infections, reinforcing the hypothesis that aggressive antibody replacement can mitigate the humoral deficiency induced by BCMA-directed BsAb therapy (8, 19). Furthermore, differences in prior BCMA-directed BsAb therapy exposure(0% in MajesTEC-1 vs. up to 53% in RWE cohorts) may contribute to variability in patient characteristics and infection risk profiles, although this relationship could not be directly established in the current analysis (2, 8, 11, 19, 20).

However, because of the limited number of studies and the heterogeneous reporting of IVIG use, our study was unable to perform a formal subgroup analysis to directly evaluate its protective effect. Nevertheless, the observed variation in IVIG utilization across studies suggests that prophylactic immunoglobulin replacement may play a critical role in mitigating infection risk during teclistamab therapy. Future prospective studies should systematically evaluate the protective impact of IVIG through controlled comparisons.

From a biological perspective, the high infection burden is intrinsically linked to teclistamab’s mechanism of action. By targeting BCMA, teclistamab induces profound depletion of both malignant and normal long-lived plasma cells, resulting in rapid-onset and persistent hypogammaglobulinemia (8). Unlike chimeric antigen receptor (CAR) T-cell therapy, which is typically administered as a one-time intervention, teclistamab requires continuous dosing, leading to sustained impairment of humoral immunity. Nevertheless, despite the increased infection risk, teclistamab offers several advantages compared with CAR-T therapy, including immediate availability, the absence of manufacturing delays, and the ability to treat patients who are not eligible for CAR-T therapy.

Another clinically relevant aspect is the temporal pattern of infection occurrence during teclistamab therapy. Unfortunately, most included studies reported only cumulative infection incidence without providing detailed time-stratified data (e.g., 0–30 days, 30–100 days, and late infections). However, previous analyses of the MajesTEC-1 trial suggest that early infections are often related to treatment initiation and immune perturbation, whereas later infections are more strongly associated with sustained hypogammaglobulinemia and cumulative immunosuppression. Consistent with this, the median onset of the first infectious event in real-world cohorts was observed around 1.5 to 2 months (46–60 days) after treatment initiation. This underscores that the infectious burden of BCMA-directed bispecific antibodies is both cumulative and time-dependent, as the lower rates in RWE cohorts (typically 3.5–5.5 months follow-up) compared to the MajesTEC-1 trial (14.1 months) likely reflect the shorter observation window. Future studies with time-dependent reporting will be crucial to better understand these dynamics.

This immunological impairment is reflected in the high incidence of infectious complications observed across studies. Regarding the etiology of infections, the included studies consistently reported a predominance of bacterial respiratory infections, particularly pneumonia. By integrating detailed etiological data from five major cohorts (N = 714), our analysis provides a granular view: bacterial and viral infections remained the primary drivers, together accounting for over 90% of events across studies, with a notable incidence of opportunistic fungal infections (ranging from 0.6% to 8%). Viral infections, including COVID-19 and cytomegalovirus reactivation, were also frequently observed, while opportunistic fungal infections such as Pneumocystis jirovecii pneumonia (PJP) were less common but clinically significant. This distribution likely reflects the profound impairment of humoral immunity induced by BCMA-targeted therapy, which predisposes patients to both typical respiratory pathogens and opportunistic infections (8, 19).

Notably, infection-related mortality reached up to 7.3%, with COVID-19 pneumonia emerging as a leading cause of death, underscoring the inability of these patients to mount effective antibody responses even after vaccination (2, 8, 11, 19, 20). It should also be acknowledged that the MajesTEC-1 trial was conducted during the COVID-19 pandemic, which may partly explain the relatively high proportion of COVID-19–related infections and mortality observed in this cohort.

Given this risk, the implementation of robust preventive strategies should be considered essential. Our findings, consistent with Mohan et al. (2024), suggest that IVIG replacement is a cornerstone of management. However, current practice remains fragmented. There is an urgent need to transition from “reactive” replacement to “standardized” prophylaxis. We propose that a unified clinical protocol, incorporating mandatory monthly IgG monitoring and pre-emptive IVIG supplementation for patients with IgG levels <400 mg/dL, could significantly standardize care and reduce the incidence of Grade ≥3 events (8, 19). In addition, primary prophylaxis against PJP and herpes zoster should be regarded as standard of care (8, 19). The “learning curve” effect observed in RWE suggests that as clinicians gain experience with BCMA-directed bispecifics, earlier recognition of immune dysfunction and timely dose modifications may preserve therapeutic efficacy while minimizing lethal toxicities.

This study has several limitations. First, the number of included studies remains limited, reflecting the recent regulatory approval of teclistamab. Second, the retrospective nature of the real-world evidence cohorts introduces potential selection and reporting biases. Third, despite subgroup and sensitivity analyses, the substantial heterogeneity observed (I²>70%) suggests that important patient-level factors, such as prior BCMA exposure, baseline immunoglobulin levels, and comorbidity burden, could not be fully accounted for in this study-level meta-analysis. Fourth, because teclistamab has been predominantly evaluated in single-arm clinical trials and observational cohorts lacking appropriate comparator arms, we employed a single-arm meta-analysis of pooled incidence as the most methodologically appropriate approach to quantify infection burden under the current evidence landscape. In addition, formal assessment of publication bias was not feasible due to the limited number of included studies, which represents an inherent limitation of early meta-analyses in rapidly evolving therapeutic fields.

### 4.1 Future perspectives

Despite these limitations, our findings provide a foundation for several important questions to be addressed in future investigation. First, whether the route of administration influences long-term infection risk warrants further study. Subcutaneous administration of BCMA-directed bispecific antibodies has been associated with improved tolerability and lower rates of acute toxicities, but its impact on cumulative immunosuppression and infection susceptibility remains unclear. Second, given that infection risk appears to be cumulative with continuous BCMA engagement, fixed-duration or response-adapted treatment strategies represent a promising approach to potentially reduce long-term infectious morbidity while preserving efficacy (21, 22). Finally, future prospective studies incorporating longitudinal immunological monitoring may help identify patients at highest risk for severe infections and inform more individualized preventive strategies.

In conclusion, while teclistamab represents a transformative treatment option for heavily pretreated RRMM, it is associated with a substantial and clinically meaningful infectious burden. Optimizing patient outcomes will require a careful balance between achieving deep hematologic responses and implementing vigilant, standardized infection surveillance and prevention strategies. These findings should inform clinical guidelines and institutional protocols for infection prevention in patients receiving BCMA-directed bispecific antibodies. Future prospective studies should focus on defining optimal IVIG replacement schedules, refining antimicrobial prophylaxis strategies, and identifying biomarkers that can predict patients at the highest risk for life-threatening infections.

## Data Availability

The original contributions presented in the study are included in the article/**Supplementary Material**. Further inquiries can be directed to the corresponding author.
