# Supplementary material for: Infection risk associated with teclistamab in relapsed/refractory multiple myeloma: a systematic review and meta-analysis of clinical trial and real-world evidence
Source: Front Immunol. 2026 Apr 30;17:1804838. doi: 10.3389/fimmu.2026.1804838 (PMC13171767; doi:10.3389/fimmu.2026.1804838)
Supplement: Supplementary file 1 [file DataSheet1.pdf]

Supplementary Figure S1. Leave-one-out sensitivity analysis for any-grade infections.

Sequential exclusion of individual studies confirms the stability of the pooled incidence estimate, with no single study disproportionately influencing the final results.

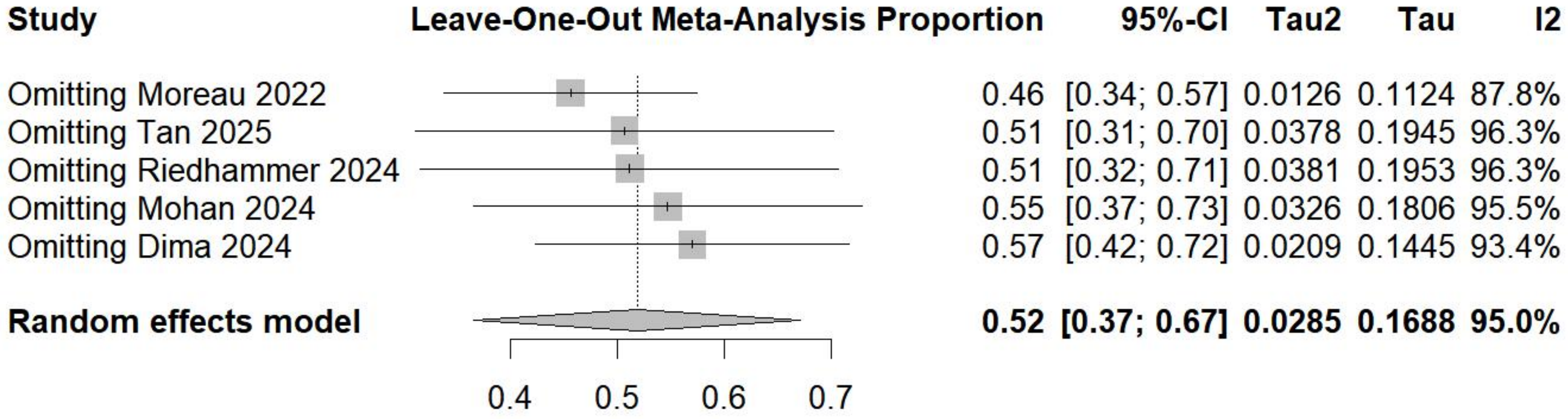

Supplementary Figure S2. Leave-one-out sensitivity analysis for grade ≥3 infections.

Stability of severe infection estimates was confirmed. Pooled incidence remained robust even after the exclusion of the high-incidence MajesTEC-1 trial.

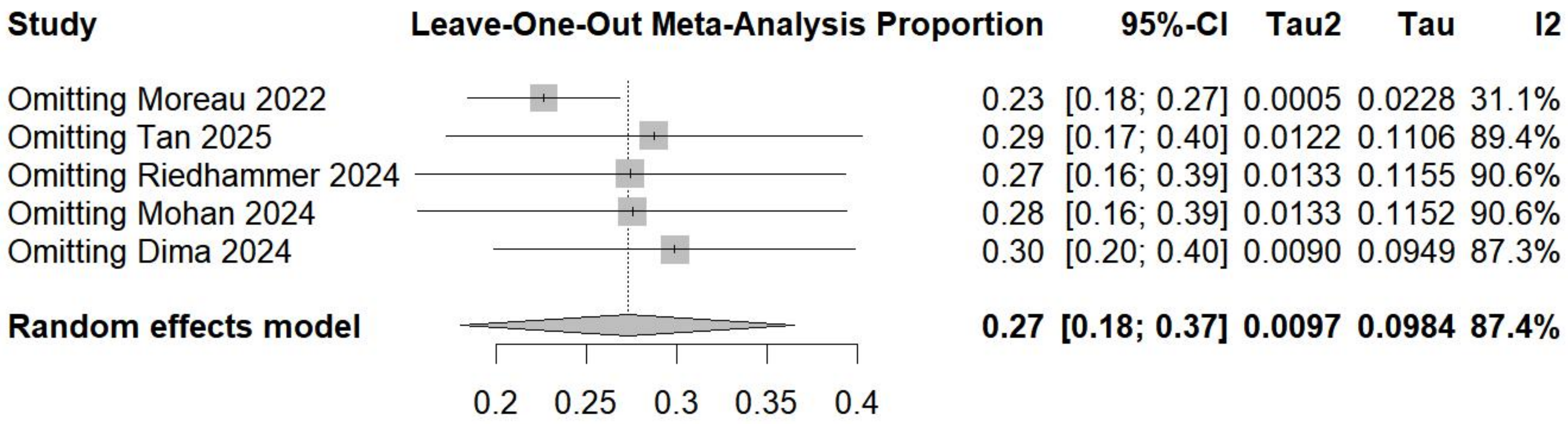

Supplementary Table S1. Detailed JBI quality assessment for each study.

The comprehensive 10-item checklist scoring for all included studies, providing the detailed rationale for the "Fair" or "Good" quality ratings.

	1. Inclusion criteria	2. Condition measurement	3. Identification methods	4. Consecutive inclusion	5. Complete inclusion	6. Demographics	7. Clinical info	8. Outcomes reporting	9. Site demographics	10. Statistical analysis
Moreau et al. 2022	Yes	Yes	Yes	Yes	Yes	Yes	Yes	Yes	Yes	Yes
Tan et al. 2025	Yes	Yes	Yes	Yes	Yes	Yes	Yes	Yes	Unclear	Yes
Riedhammer et al. 2024	Yes	Yes	Yes	Yes	Yes	Yes	Yes	Yes	Unclear	Yes
Mohan et al. 2024	Yes	Yes	Yes	Yes	Yes	Yes	Yes	Yes	Unclear	Yes
Dima et al. 2024	Yes	Yes	Yes	Unclear	Unclear	Yes	Yes	Yes	Unclear	Yes
